# Nephroprotective activity of naringin against chemical-induced toxicity and renal ischemia/reperfusion injury: A review

**DOI:** 10.22038/AJP.2022.19620

**Published:** 2022

**Authors:** Negin Amini, Maryam Maleki, Mohammad Badavi

**Affiliations:** 1 *Department of Physiology, School of Medicine, Ahvaz Jundishapur University of Medical Sciences, Ahvaz, Iran*; 2 *Persian Gulf Physiology Research Center, Medical Basic Sciences Research Institute, School of Medicine, Ahvaz Jundishapur University of Medical Sciences, Ahvaz, Iran*; 3 *Department of Physiology, School of Medicine, Ilam University of Medical Sciences, Ilam, Iran*

**Keywords:** Kidney, Naringin, Oxidative stress, Toxicity

## Abstract

**Objective:**

The kidney is well-known as the vital organ which is responsible for maintaining body homeostasis and secretion of toxic metabolites. Renal injury is accompanied by oxidative stress which results in cellular apoptosis, lipid peroxidation, and reduction of antioxidant levels. Plant extracts and their phytoconstituents, owing to free radical scavenging properties, seem to be valuable against modern synthetic and chemical drugs. Naringin is a flavonoid present in citrus fruits with pharmacologic effects including antioxidant, anti-inflammatory, and anti-apoptotic properties. This review summarizes the renoprotective effects of naringin and discusses mechanisms of its action against renal injury.

**Materials and Methods:**

For this paper, original subject-related articles published up to October 2020 have been reviewed in the databases, including PubMed, Scopus, and Web of Science, and Google Scholar.

**Results:**

Naringin increases antioxidant enzyme activity, and glutathione content, reduces lipid peroxidation and inhibits inflammatory cytokines. In the molecular investigation, naringin activates the *Nrf-2* signaling, prevents apoptosis signaling, and inhibits the autophagy pathway. Besides, naringin could protect the kidney through modulating *microRNA-10a* in the kidney tissue in an acute kidney injury model.

**Conclusion:**

This review recommends that naringin can be considered a promising candidate to treat kidney dysfunction induced by oxidative stress in the future.

## Introduction

The kidney is a vital organ that is responsible for different functions in the body, such as the maintenance of body homeostasis, as well as water and electrolyte balance, the exertion of toxic metabolites, and regulation of glomerular filtration (Bello-Reuss and Reuss, 1983 ▶; Ferguson et al., 2008 ▶). In this regard, maintaining kidney health is essential, since kidney dysfunction leads to water-electrolyte imbalance, accumulation of metabolites, resulting in harmfull consequences (Di Lullo et al., 2019 ▶). Environmental toxins, medicinal agents, and ischemia-reperfusion (I/R) have been demonstrated to cause acute kidney injury (AKI) (Abd Elmonem et al., 2018 ▶; Adil et al., 2015 ▶; Amini et al., 2019b ▶). Oxidative stress is one of the main contributors to kidney injury that is associated with lipid peroxidation, depletion of antioxidant enzymes, cell apoptosis, mitochondrial dysfunction, and finally, cell death (Araujo and Welch, 2006 ▶; Ratliff et al., 2016 ▶). 

Nowadays, preventive or supplementary medicine is applied to treat various chronic diseases (McDonough and Doucette, 2003 ▶) and plant-based dietary nutrients seem to be unique alternatives in the prevention and treatment of diseases, due to the side effects of chemical drugs and the lack of protective kidney medication in medicine (Martin and Appel, 2009 ▶; Yadav et al., 2020 ▶). It was confirmed that herbal-based nutrients, especially polyphenolic compounds (flavonoids, anthocyanins, and phenolic acids), possess potential health benefits (Kandemir et al., 2017 ▶; Martin and Appel, 2009 ▶). Flavonoids are the most potent antioxidants among polyphenolic compounds (Martin and Appel, 2009 ▶), which through antioxidant and inflammatory properties, can influence several molecular signaling pathways to protect organ function (Chen et al., 2018a ▶; Chen et al., 2018b ▶). *Citrus* plants are well-known as polyphenolic compounds, and naringin is one of the main flavonoids extracted from their fruits (Tripoli et al., 2007 ▶). Naringin (C_27_H_32_O_14_, molecular weight: 580.4 g/mol) is a flavanone glycoside derived from naringenin (Zhang et al., 2014 ▶). It is found in grapes and citrus fruits and has a bitter taste of citrus juices (Chtourou et al., 2015 ▶). In the intestine, gut microflora breaks down naringin to its aglycon, naringenin; then, it is absorbed through portal blood (Choudhury et al., 1999 ▶).

In experimental studies, it has been confirmed that following oral consumption and absorption through portal blood, naringin undergoes vast metabolisms which can control the bioavailability of naringin in the plasma. Naringin is eliminated via bile excretion by the liver (Alam et al., 2014 ▶; Tsai and Tsai, 2012 ▶). Oral acute and subchronic administrations of naringin indicate non-toxic effects in experimental studies (Li et al., 2013 ▶). In a rat model, it has been indicated that the median lethal dose (LD50) of naringin was 2000 mg/kg (Bo et al., 2013 ▶; Li et al., 2014 ▶). In numerous studies, antioxidant, anti-inflammatory, anti-apoptosis, metal chelating, and anti-carcinogenic properties of naringin have been proven (Camargo et al., 2012 ▶; Chtourou et al., 2016 ▶; Singh et al., 2004a ▶). Considering several pharmacologic properties, high availability, and low cost, naringin could be considered a potential candidate for renal diseases. 

**Figure 1:**
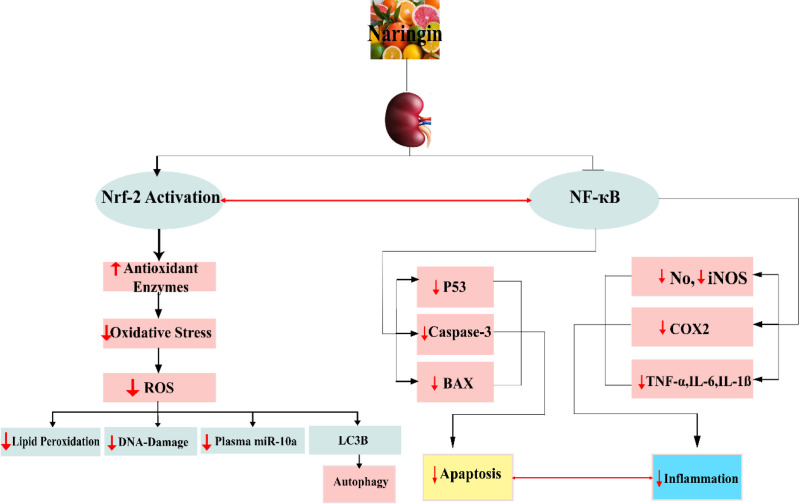
Naringin produces potential protective effect against AKI due to its antioxidative, anti-inflammatory, and antiapoptotic properties via upregulation of *Nrf-2* and downregulation of *miR-10a* and *NF-Ƙβ*

The present review was done to determine the beneficial roles of naringin and its mechanisms of action in AKI (Naringin effects are summarized in Figure 1).

## Materials and Methods

For this study, original subject-related articles published up to October 2020 have been reviewed in the databases, including PubMed, Scopus, and Web of Science, and Google Scholar. The search was performed using these keywords: “Naringin”, “kidney”, “nephrotoxicity”, “renal ischemia-reperfusion injury”, and “protective.” All articles that evaluated the effect of naringin on nephrotoxicity were included in this review (Table 1). 

## Results

**The role of naringin in cisplatin nephrotoxicity**

Cisplatin is one of the most common and potent chemotherapy drugs used for malignancy treatment (Pabla and Dong, 2008 ▶). The action mechanism of cisplatin for organ dysfunction is through oxidative stress (Pabla and Dong, 2008 ▶). Nowadays, the use of natural products is considered an alternative treatment (Martin and Appel, 2009 ▶). Elmoniem et al*.* showed that naringin improves kidney function by reducing malondialdehyde (MDA), tumor necrosis factor-α (TNF-α), cyclooxygenase-2 (COX-2), myeloperoxidase (MPO), and enhancing glutathione (GSH) (Taha et al., 2018 ▶). Chtourou and his colleagues have recommended that naringin exerts nephroprotective effects against cisplatin-induced toxicity by reducing blood urea nitrogen (BUN), creatinine (Cr), urea, uric acid, MDA, nuclear factor-kappa-β (NF-Ƙβ), caspase-3, and reactive oxygen species (ROS) levels, as well as by increasing GSH, catalase (CAT), superoxide dismutase (SOD), glutathione peroxidase (GP_X_), and glutathione transferase (GST) due to eliminating free radicals (Abd Elmonem et al., 2018 ▶; Chtourou et al., 2016 ▶).

**The role of naringin in acetaminophen nephrotoxicity**

Acute overdose or extreme chronic administration of acetaminophen (N-acetyl-p-aminophenol, APAP), leads to extreme side effects, including renal toxicity (Gum and Cho, 2013 ▶; Isik et al., 2006 ▶). APAP led to the metabolic activation of N-acetyl-p-benzoquinone imine (NAPQI). The overdose of APAP leads to a reduction in GSH content (Isik et al., 2006 ▶). Moreover, the overdose of APAP saturates the detoxication pathways of APAP due to sulfation insufficiency. Consequently, GSH depletion results in the accumulation of APAP, intensifying cellular oxidative stress (Jollow et al., 1973 ▶).

**Table 1:** Renoprotective effects of naringin against acute kidney injury

**Study design (model), dose of Narginin (administration route, duration)**	**Model of AKI **	** Parameters **	**Results**		**References**
***In vivo*** ** (rat),** **20, 40, and 80 mg/kg (orally, 14 days) **	APAP, 700 mg/kg	SOD, GSH BUN, Cr, NO, MDA, KIM	Increase Decrease		
***In vivo*** ** (rat)** **20 mg/kg (orally, 4 weeks) **	APAP, 0.5 gr/kg	GSH, GST, GPx, SOD Cr, Urea, Uric acid, MDA	Increase Decrease		
***In vivo*** ** (rat), 80 mg/kg (orally, 7 days) **	Cispaltin, 7 mg/kg	GSH MDA, Cr, Urea, COX-2, NO, MPO, TNF-α	Increase Decrease		
***In vivo*** ** (rat), 25, 50, and 100 mg/kg (orally, 5 weeks) **	Cispaltin, 5 mg/kg	The enzymes cascade, C_cr_ Lipide peroxidative markers, BUN, Cr, Uric acid, NO, TNF-α, p53, NF-ƘB, iNOS, p53, Caspas-3	Increase Decrease		
***In vivo*** ** (rat), 80 mg/kg** **(orally, 14 days) **	Cispaltin 7 mg/kg	GSH Urea, Cr, MDA, COX-2, MPO, NO, TNF-α	Increase Decrease		
***In vivo*** ** (rat), 100, 200, and 400 mg/kg (orally, 60 min) **	Glycerol, 8 ml/kg	GSH, CAT, SOD, GR, C_cr_, C_urea _ BUN, Cr, MDA	Increase Decrease		
***In vivo*** ** (rat),** **100 mg/kg (IP, 14 days)**	5-FU, 20 mg/mg	SOD, GSH BUN, Cr, TNF-α, IL-6, IL-1α, Lipide peroxidative marker	Increase Decrease		
***In vivo*** ** (rat)** **20,40, and 80 mg/kg (orally, 20 days)**	Nickel, 20 mg/kg	The enzymes cascade, Urine output Lipide peroxidative markers, Urea, Cr, Nickel toxicity, Uric acid	Increase Decrease		
***In vivo*** ** (rat), 20,40, and 80 mg/kg (orally, 28 days) **	Sodium arsenite, 5 mg/kg	Urine Cr, SOD, GSH, Crc Cr, BUN, Uric acid, NO, MDA, KIM-1, TNF-α, Caspase-3, TGF-β	Increase Decrease		
***In vivo*** ** (rat), 50 and 100 mg/kg (orally, 7 days) **	Gentamicin, 120 mg/kg	GSH, GPx, GR, GST, SOD, CAT, and Vitamin C, NDH, SDH, COX Cr, BUN, Lipide peroxidative markers, TNF-α, IL-6, MPO, p53, NF-ƘB, Caspase-3, Caspase-9, BAX	Increase Decrease		
***In vivo*** ** (rat), 50 and 100 mg/kg (orally, 7 days) **	MTX, 20 mg/kg	SOD, GPx, CAT, GSH Urea, Cr, MDA, TNF-α, IL-1β, IL-6, NF-Ƙβ, iNOS, COX-2, Caspase-3, LC3B	Increase Decrease		
***In vivo* (rat), 40 mg/kg (orally, 21 days) **	CsA, 25 mg/kg	The antioxidant cascade, HO-1 Lipide peroxidative markers	Increase Decrease		
***In vivo *** **(rat), 100, 200, and 400 mg/kg (Orally, 1 hr)**	Fe–NTA, 8 mg/kg	CAT, GR, SOD, GSH BUN, Cr, MDA	Increase Decrease		
***In vivo*** ** (rat), 50, and 100 mg/kg (orally, 7 days) **	CYP, 200 mg/kg	SOD, GPx, CAT, GSH Urea, Cr, MDA, NF-Ƙβ, TNF-α, IL-1β, IL-6, iNOS, LC3B, Caspase-3, COX-2, 8-OHdG (marker of oxidative DNA damage)	Increase Decrease		Caglayan et al. (2018)
***In vivo*** ** (rat), 400 mg/kg, (orally, 24 hr)**	Renal I/R	C_cr_, GSH, CAT, SOD BUN, Cr, MDA	Increase Decrease		
***In vivo*** ** (rat), 100 mg/kg** **(IP, 7 days)**	Renal I/R	C_cr_, RBF, SOD, GPx, BCL-2 BUN, Cr, FE_Na, _miR-10a, BAX, Caspase-3	Increase Decrease		
***In vivo*** ** (rat), 100 mg/kg (IP, 7 days) **	Renal I/R	CAT, TAC, Nrf-2 MDA	Increase Decrease		
***In vivo*** ** (rat), 25,50, and 100 mg/kg (orally, 28 days) **	STZ, 40 mg/kg	PPARƳ, HSP-72, SOD, GSH NF-ƘB, TBARS	Increase Decrease		
***In vivo*** ** (rat), 20,40, and 80 mg/kg (IV, 12 weeks) **	STZ, 60 mg/kg HG-induced podocytes	SOD, GSH-Px BUN, Cr, Urinary Protein, MDA, ROS, Caspase-3, NOX4	Increase Decrease		

BUN: Blood urea nitrogen; Cr: Creatinine; KIM-1: Kidney injury molecule-1; GSH: Glutathione; GST: Glutathione transferase; GPx: Glutathione peroxidase; SOD: Superoxide dismutase; MDA: Malondialdehyde; TBARS: Thiobarbituric acid reactive substances; COX-2: Cyclooxygenase-2; NO: Nitric oxide; MPO: Myeloperoxidase; TNF-α: Tumor necrosis factor-α; NF-ƘB: Nuclear factor-kappa-B; GR: Glutathione reductase; IL-6: Interleukin-1α; IL-1α: Interleukin-1α; CAT: Catalase; BAX: Bcl-2 associated x protein; BCL-2: B-cell lymphoma 2; RBF: Renal blood flow; miR-10a: MicroRNA-10a; Nrf-2: Nuclear factor erythroid 2-related factor 2; LC3B: light chain 3B; HO-1: Heme oxygenase1; TGF-β: Transforming growth factor beta;  ROS: Reactive oxygen species; NDH: NADH dehydrogenase; SDH: Succinate dehydrogenase; FE_Na : _Fractional excretion of sodium; PPAR: Peroxisome proliferator-activated receptor-gamma; HSP-72: Heat-shock proteins; NOX4: NADPH oxidase; 5-FU: 5-fluorouracil; APAP: N-acetyl-p-aminophenol; MTX: Methotrexate; CsA: Cyclosporine; Fe–NTA: Ferric nitrilotriacetic acid; CYP: Cyclophosphamide; I/R: Ischemia/reperfusion; STZ: Streptozotocin

Naringin inhibited oxidative stress induced by APAP by decreasing MDA and augmenting antioxidant enzymes, including SOD, GP_X_, GST, and GSH content in the kidney tissue. Likewise, naringin pretreatment ameliorated plasma BUN and Cr levels, indicating improved kidney function (Adil et al., 2016 ▶; Ahmed et al., 2019 ▶). 

Histopathological changes, including glomerular hypertrophy, congestion of renal vessel, pyknosis, and karyorrhexis, as well as markers of inflammation were appeared in APAP-induced kidney toxicity in rats, indicating renal cell death, which was relieved via an antioxidant effect of naringin (Adil et al., 2016 ▶; Ahmed et al., 2019 ▶).

Kidney injury molecule-1 (KIM-1) is considered as a new prognostic biomarker, which plays a pivotal role in the manifestation of renal injury (Ichimura et al., 2008 ▶; Visnagri et al., 2015 ▶). Oxidative stress induced by APAP elevated *KIM-1* expression in kidney tissue, whereas naringin administration modified enhancement of renal *KIM-1* expression in APAP-exposed rats (Adil et al., 2016 ▶). Taken together, these findings recommended that the beneficial effects of naringin might be mediated, at least in part through increasing the antioxidant defense system and suppressing oxidative stress. 

**The role of naringin in cyclophosphamide nephrotoxicity**

Cyclophosphamide (CYP) is an anti-neoplastic medicine which is extensively used for the treatment of numerous diseases (Sinanoglu et al., 2012 ▶). Histopathology alterations in the kidney, depletion of GSH, and enhanced renal MDA level are caused by CYP (Sugumar et al., 2007 ▶). 

An experimental study reported that CYP activated inflammatory, apoptotic, and autophagic pathways. CYP leads to reduced antioxidant capacity, including GSH, and enhances lipid peroxidation, which produces oxidative stress; the lack of GSH content has been ascribed to the direct conjugation of CYP and its metabolites with an SH– group (Rehman et al., 2012 ▶). According to an experimental study, naringin recovered the levels of non-enzymatic (GSH) and enzymatic antioxidants (SOD, GP_X_, and CAT). In addition, CYP-induced inflammatory responses were decreased, indicating the renoprotective effect of naringin due to removing free radical oxygen. Moreover, the administration of naringin considerably decreased the expression of 8-OHdG which is identified as an index of oxidative DNA damage. Also, CYP-induced the apoptotic and autophagic pathways by enhancing *caspase-3* expression and *light* *chain 3B (LC3B)* level, indicating that naringin protects CYP-induced nephrotoxicity through suppressed apoptotic and autophagy pathways (Rehman et al., 2012 ▶). 

**The role of naringin in 5-fluorouracil nephrotoxicity**

5-fluorouracil (5-FU) is categorized as an anti-metabolic agent which is generally used for the treatment of multiple cancers (Longley et al., 2003 ▶). 5-FU leads to overproduction of ROS like the other chemotherapeutic agents and causes harmful outcomes and toxicity in kidney and liver tissues (Gelen et al., 2018 ▶; Longley et al., 2003 ▶). Depletion of antioxidant capacity elevated MDA content, and kidney dysfunction was reported in rats exposed to 5-FU (Gelen et al., 2018 ▶). 

Administration of naringin significantly attenuated 5-FU-induced oxidative stress by modulating cellular antioxidant capacity. Naringin pretreatment recovered SOD and GSH, resulting in a considerable reduction in kidney MDA levels and improvement of kidney function. Moreover, inflammatory mediators' levels, including interleukin-1α (IL-1α), TNF-α, and interleukin-6 (IL-6) were inhibited with naringin treatment, which confirms the anti-inflammatory effect of naringin following 5-FU nephrotoxicity (Gelen et al., 2018 ▶).

**The role of naringin in methotrexate nephrotoxicity**

Methotrexate (MTX) has been used to treat multiple cancers, but due to numerous side effects such as nephrotoxicity, its usage has been limited in clinics (Kalemci et al., 2015 ▶). Like the other chemotherapeutic drugs, not only MTX depletes the antioxidant enzyme systems but it also could produce ROS (Kandemir et al., 2017 ▶). Among the different agents that have diminished the anticancer drug-induced oxidative stress, natural products have been received substantial interest, owing to their uniqueness (Benzer et al., 2012 ▶; Gülcin, 2012 ▶). Cytosolic nicotinamide adenosine diphosphate (NADP)-dependent dehydrogenases and NADP malic enzymes are suppressed by MTX to decline the amount of NADPH in the cells (Abdel-Raheem and Khedr, 2014 ▶). In physiologic situations, NADPH converts glutathione into reduced glutathione which is essentially a non-enzymatic antioxidant to protect cells against oxidative damage (Bubici et al., 2006 ▶). MTX-induced renal toxicity reduces the cellular defense system via diminishing GSH content and antioxidant enzyme levels, which result in cellular damage by changing macromolecular structures including membrane lipids, proteins, and nucleic acids. Thus, the enhancement of lipid peroxidation can occur (Kandemir et al., 2017 ▶). Caspase is the main effector in apoptotic cell death, and caspase-3 has an essential function in activating cell death signals among caspases (Jänicke et al., 1998 ▶). MTX provoked apoptosis and autophagy via increasing *caspase-3* and *LC3B* levels (Kandemir et al., 2017 ▶). 

NF-ƘB is activated by oxidative stress situations, resulting in the generation of inflammatory mediators by MTX (Kandemir et al., 2017 ▶). Naringin administration was able to increase GSH content and antioxidant enzymes, which was accompanied by lipid peroxidation inhibition in the kidney tissue. Additionally, naringin reduced *LC3B* and *caspase-3* levels that indicate a renoprotective effect of naringin through anti-autophagic and anti-apoptotic properties in MTX nephrotoxicity (Kandemir et al., 2017 ▶). 

**The role of naringin in cyclosporine (A) nephrotoxicity**

Cyclosporine (CSA) is considered an immunosuppressant drug to prevent the rejection of organs in transplantation (Busauschina et al., 2004 ▶). ROS have been shown as detrimental agents in various renal injuries, including nephrotoxicity (Anjaneyulu et al., 2003 ▶). CSA-induced oxidative stress results in structural kidney damage and a reduction of glomerular filtration rate (GFR) confirmed by histopathological findings (Chandramohan and Parameswari, 2013 ▶). Nowadays, many researchers have been attracted to citrus flavonoids owing to their beneficial role in health (Anjaneyulu et al., 2003 ▶). Naringin has been able to enhance the renal enzyme and non-enzymatic antioxidant levels and reduce lipid peroxidation. The detailed mechanism of ameliorating CSA-induced nephrotoxicity by naringin, is unknown (Chandramohan and Parameswari, 2013 ▶). 

The heme oxygenase (HO) enzyme system has two significant isoforms: HO-1 and HO-2. HO-1 induction has beneficial effects in diseases, and its protective properties are manifested by its anti-inflammatory, anti-apoptotic, and antioxidant effects (Nath, 2006 ▶). The mRNA and protein level of *HO-1*, which catalyzes the rate-limiting part of heme degradation, markedly reduced following treatment with CSA in rats (Chandramohan and Parameswari, 2013 ▶).

In the molecular investigation, naringin administration caused a significant elevation of mRNA and protein levels of *HO-1* in renal tissue, implying cytoprotective effects of naringin in a CSA-induced toxicity model (Chandramohan and Parameswari, 2013 ▶). 

**The role of naringin in gentamycin nephrotoxicity**

Gentamicin (GM) is identified as one of the most common aminoglycoside antibiotics. Owing to the low cost, low-level resistance, and easy availability, it is administered for the treatment of infections resulting from Gram-negative aerobes (Ali et al., 2011 ▶; Negrette-Guzmán et al., 2013 ▶). Despite its salutary effects, profound implications like nephrotoxicity are the limiting factors for using aminoglycosides (Sun et al., 2013 ▶). Experimental studies recommend that ROS play a role in lipid peroxidation and reduced enzymatic antioxidant system in the nephrotoxicity model including GM-induced nephrotoxicity (Balakumar et al., 2010 ▶; Lee et al., 2012 ▶). GM-induced ROS generation is an important key to cause ROS mitochondrial production and subsequently, mitochondrial dysfunction, which triggers apoptosis signaling (Morales et al., 2010 ▶; Juan et al., 2007 ▶; Bae et al., 2014 ▶). 

 Recently, naringin received considerable attention in treating various diseases as a free radical scavenger (Tripoli et al., 2007 ▶). GM-induced nephrotoxicity results in ROS overproduction that damages protein molecules and degrades the membrane-bound phospholipids, leading to lipid peroxidation, while naringin treatment changed this enhanced lipid peroxidation (Sahu et al., 2014 ▶). 

Antioxidant enzymes SOD, CAT, GPx, GR (glutathione reductase), and GST activities, and non-enzymatic antioxidant levels (GSH and vitamin C) were remarkably decreased in GM nephrotoxicity. A reduction of GSH content is due to overproduction of GM-induced free radicals, or increased use of GSH to protect proteins containing -SH group against free radicals (Sahu et al., 2014 ▶).

Furthermore, the extensive formation of free radicals contributes to reducing SOD and CAT activities in GM-induced nephrotoxicity. However, naringin administration improved antioxidant enzyme activity. These observations confirm the hypothesis that the beneficial effects of naringin are related to enhancing the antioxidant defense system (Sahu et al., 2014 ▶). 

Mitochondrial dysfunction is considered one of the mechanisms of GM-induced nephrotoxicity; in fact, ROS overproduction leads to kidney morphological and functional changes (Sahu et al., 2014 ▶). Administration of naringin ameliorated mitochondrial function, showing enhancement of the activities of mitochondrial respiratory enzymes (Sahu et al., 2014 ▶). Mitochondrial membrane permeability, associated with GM nephrotoxicity, results in the activation of apoptosis signaling (Sahu et al., 2014 ▶). 

However, naringin administration reduced the level of *cleaved caspase-3*, *Bcl-2 associated x protein (Bax), and p53* protein expression and promoted the *B-cell lymphoma 2 (Bcl-2)* protein level (Sahu et al., 2014 ▶). Thus, naringin could affect intrinsic/extrinsic apoptotic signaling pathway through inhibition of *p53, Bax*, and elevation of *Bcl-2* expression in the renal tissue of GM-administered rats.

Other findings indicated that the enhanced oxidative stress was reduced by naringin, which could be owing to naringin antioxidant, anti-inflammatory, anti-apoptosis, and mitochondrial protection properties (Sahu et al., 2014 ▶). 

**The role of naringin in ferric nitrilotriacetate nephrotoxicity**

Ferric nitrilotriacetic acid (Fe-NTA) is an iron chelating that leads to a significant enhancement of lipid peroxidation and renal oxidative injury, ultimately inducing proximal tubular necrosis (Singh et al., 2004a ▶). Fe-NTA induced a remarkable kidney oxidative stress indicated by increased MDA and reduced renal CAT, SOD, and GR levels (Singh et al., 2004a ▶).

An experimental study reported that pretreatment with a single dose of naringin decreased levels of BUN and Cr in Fe-NTA nephrotoxicity. Naringin pretreatment significantly ameliorated kidney dysfunction and morphological alterations, diminished MDA, and recovered the depleted renal antioxidant enzymes (Singh et al., 2004a ▶). 

**The role of naringin in glycerol-induced nephrotoxicity**

AKI induced by glycerol is associated with tremendous oxidative stress which results in kidney vasoconstriction, direct cytotoxicity, and cast formation. Administration of naringin amended lipid peroxidation and increased the level of antioxidant enzymes and GSH in the kidney in glycerol-treated rats (Singh et al., 2004b ▶). Naringin possesses the potential antioxidant properties which recover kidney dysfunction, thereby increasing the antioxidant capacity and decreasing lipid peroxidation in the tissue (Singh et al., 2004b ▶).

**The role of naringin in heavy metal nephrotoxicity**

Pollution caused by heavy metals is the most common micro-element pollutants in the environment. Toxic metals, including arsenic, cadmium, lead, and nickel, through overproduction of ROS, contribute to disturbing antioxidant system balance which ultimately leads to the undesirable effects on human health (Adil et al., 2015 ▶; Amudha and Pari, 2011 ▶; Nejabat et al., 2017 ▶). 

**The role of naringin in nickel nephrotoxicity**

Nickel is well-known as an environmental pollutant that is used in industry. Cigarette smoking and ingesting contaminated foodstuffs cause the transportation of nickel to various organs, including the kidney that leads to renal cell injury (De Brouwere et al., 2012 ▶; Edelman and Roggli, 1989 ▶; Gitlitz et al., 1975 ▶). Nickel toxicity is associated with oxidative stress, alterations of histopathology, a significant increase of plasma BUN and Cr levels, and reduction of Cr clearance (Amudha and Pari, 2011 ▶). 

The effects of naringin in nickel-induced renal toxicity have been reported by Amudha and Pari (Amudha and Pari, 2011 ▶). Administration of naringin led to ameliorating kidney function and reducing lipid and protein peroxidation indexes, indicating the free radical scavenging properties (Amudha and Pari, 2011 ▶). Moreover, a significant decrease in nickel accumulation in the kidney tissue was reported following naringin treatment, showing that the hydroxyl groups of naringin could bind to nickel, raise the excretion of nickel and prevent the accumulation of nickel in kidney tissues; so, naringin decreases toxic effects of nickel (Amudha and Pari, 2011 ▶). 

**The role of naringin in arsenic nephrotoxicity**

Arsenic is well-known as a toxic agent in the environment, causing harmful effects in organs of animals and humans (Kimura et al., 2006 ▶; Liu et al., 2000 ▶; Singh and DuMond, 2007 ▶). Oxidative stress, inflammatory factors, and apoptosis pathways are mechanisms for arsenic toxicity (Adil et al., 2015 ▶). 

The beneficial effects of naringin on sodium arsenite-nephrotoxicity were investigated by Adil et al. (2015) ▶. Pretreatment with naringin remarkably enhanced SOD enzyme activity and GSH content, decreased MDA level in the kidney, and reduced plasma BUN and Cr levels (Adil et al., 2015 ▶). At the molecular level, naringin could suppress the increase of inflammatory cytokine *TNF-α, caspase-3,* and *KIM-1* in the kidney. The result of this study showed oxidonitrosative stress in renal tissue was recovered by naringin through its antioxidant, anti-apoptosis, and anti-inflammatory properties (Adil et al., 2015 ▶). 

**The role of naringin in streptozotocin nephrotoxicity**

It has been estimated that almost half the diabetic individuals have type 2 diabetes mellitus (T2DM), with the most common complications of diabetic nephropathy (DN) (Shaw et al., 2010 ▶; Wang et al., 2011 ▶). Growing evidence demonstrated that oxidative stress is considered a critical agent in the exacerbation of pathogenesis of DN (Wolf and Ziyadeh, 2007 ▶).

Streptozotocin (STZ) has been commonly utilized to create experimental models of diabetes through its cytotoxic action on pancreatic β-cells (Rodríguez et al., 2018 ▶). STZ’s toxic effect is associated with oxidative damage, inflammation, and apoptosis (Sharma et al., 2011 ▶).

Numerous studies have shown that the administration of naringin remarkably ameliorates antioxidant status in diabetic rats (Sharma et al., 2011 ▶; Zhang et al., 2017 ▶). Naringin significantly improved the altered levels of antioxidant enzymes (SOD and GP_X_) and reduced lipid peroxidation biomarkers in the kidney tissue and serum. The contents of MDA, and pro-inflammatory cytokines IL-6 and TNF-α in both serum and kidney were diminished following naringin administration in diabetic rats, indicating the inhibition of lipid peroxidation generation, inactivation of NF-ƘB, and suppression of free radical production (Sharma et al., 2011 ▶; Zhang et al., 2017 ▶). An *in vitro* study suggests that naringin inhibits ROS generation and reduces the MDA level in high glucose-treated podocytes (Zhang et al., 2017 ▶). Naringin prevented the *NF-ƘB* overexpression induced by oxidative stress in STZ rats by removing ROS, which activated this molecular pathway (Sharma et al., 2011 ▶). PPAR (peroxisome proliferator-activated receptor-gamma) is identified as a nuclear receptor that applies its action through anti-inflammatory, and antioxidant effects in glomeruli (Michalik and Wahli, 2006 ▶). Additionally, heat-shock proteins (HSP) protect cells against many stressors by potential antioxidant and anti-inflammatory effects (Chung et al., 2008 ▶). Investigation of molecular has revealed that naringin results in *PPAR, HSP-27*, and *HSP-72* overexpression in STZ-induced DN in rats (Sharma et al., 2011 ▶). Besides, *NADPH oxidase 4 (NOX4*) as the primary source of ROS production in renal cells, was downregulated by naringin in DN rats (Zhang et al., 2017 ▶). Following ROS production, the activation of caspase-3 as a key for the execution of apoptosis signaling occurs, which was repressed in the presence of naringin in STZ-induced DN in rats and HG-treated podocytes (Zhang et al., 2017 ▶).

Microscopic study of kidney tissue from STZ rats manifested hydropic changes in proximal convoluted tubules and widening of the matrix, which were improved by naringin (Sharma et al., 2011 ▶). 

To sum up, it can be recommended that the protective effects of naringin is attributed to antioxidant properties. Moreover, naringin improved renal dysfunction which occurred by upregulation of *PPARγ, HSP-72,* and *HSP-27,* suppression of NF-kβ, and anti-apoptosis effect.

**The role of naringin in renal I/R injury**

Renal I/R injury is identified as one of the most common causes of AKI (Shoskes and Halloran, 1996 ▶). During ischemia, regeneration of ROS, alteration of cell membrane permeability, and imbalance of ions occur. Although reperfusion is essential for providing ATP production, it intensifies tissue injury through ROS overproduction and apoptosis (Kalogeris et al., 2012 ▶). 

Recently, the role of naringin by free radical removal has been noted in health (Kalogeris et al., 2012 ▶). The salutary effects of naringin in renal I/R injury models have been documented (Amini et al., 2019b ▶). Naringin pretreatment remarkably recovered the levels of antioxidant and suppressed MDA level in the kidney tissue in a renal I/R model (Amini et al., 2019a ▶; b ▶). Moreover, an experimental study reported that naringin changed BUN and Cr levels in the plasma that demonstrated improvement of kidney function (Amini et al., 2019b ▶). 

Moreover, molecular analysis showed that naringin significantly down-regulated *caspase-3* and *Bax* mRNA and augmented the Bcl-2 expression level (Amini et al., 2019b ▶). Nuclear factor erythroid 2-related factor 2 *(Nrf-2)* is identified as a cell-protecting agent against stressors including ROS, and radiation that regulate the transcription of antioxidant enzyme genes (Huang et al., 2015 ▶). Naringin administration increased kidney *Nrf-2* mRNA expression in renal I/R injury, indicating cell protection due to induction of the transcription of critical antioxidant enzyme genes (Amini et al., 2019a ▶). 

*MicroRNA-10a (miR-10a)* is identified as a kidney tissue-specific microRNA, released from the tissue into the plasma following oxidative stress (Wang et al., 2012 ▶). Naringin caused a reduction in the renal I/R-induced high plasma *miR-10a* level that suggests its cytoprotective effect. These findings exhibited that naringin, in optimal concentration, exerts renoprotective effects through upregulation of *Nrf-2*, which is associated with increasing activity of antioxidant enzymes, reducing apoptosis, and downregulation of *miR-10a* level in a renal I/R injury model (Amini et al., 2019a ▶; b ▶).

## Discussion

According to the mentioned points, AKI is associated with a group of markers such as elevated BUN and Cr and inflammation responses to renal failure. Nowadays, herbal medicines, due to less side effects, are used instead of chemical medicines. In this regard, naringin which has anti-inflammatory and potential free radical scavenging effects inhibits glomerular dysfunction and renal injury. Naringin can prevent kidney injury through various pathways. Naringin has limited the oxidative stress by triggering the *Nrf-2* pathway and declined pro-inflammatory factors such as *COX-2, TNF-α, iNOS, *and apoptosis agents. Natural products could stimulate the *Nrf-2* signaling pathway to regulate the gene expression of antioxidants (Molaei et al., 2021 ▶). This review promises the potential clinical applications of naringin, after undergoing clinical trials, in the treatment and prevention of kidney disease.

## Conflicts of interest

The authors have declared that there is no conflict of interest.

## References

[B1] Abd Elmonem AT, Khalifa M, Abdel-Salam MI (2018). Nephroprotective role of naringin against cisplatin-induced nephrotoxicity. Malaysian J Med Res.

[B2] Abdel-Raheem IT, Khedr NF (2014). Renoprotective effects of montelukast, a cysteinyl leukotriene receptor antagonist, against methotrexate-induced kidney damage in rats. Naunyn Schmiedebergs Arch Pharmacol.

[B3] Adil M, Kandhare AD, Ghosh P, Venkata S, Raygude KS, Bodhankar SL (2016). Ameliorative effect of naringin in acetaminophen-induced hepatic and renal toxicity in laboratory rats: role of FXR and KIM-1. Ren Fail.

[B4] Adil M, Kandhare AD, Visnagri A, Bodhankar SL (2015). Naringin ameliorates sodium arsenite-induced renal and hepatic toxicity in rats: decisive role of KIM-1, Caspase-3, TGF-beta, and TNF-alpha. Ren Fail..

[B5] Ahmed O, Fahim H, Ahmed H, Mahmoud B, Aljohani S, Abdelazeem W (2019). The nephropreventive and antioxidant effects of navel orange peel hydroethanolic extract, naringin and naringenin in n-acetyl-p-aminophenol-administered wistar rats. Adv Anim Vet Sci.

[B6] Alam MA, Subhan N, Rahman MM, Uddin SJ, Reza HM, Sarker SD (2014). Effect of citrus flavonoids, naringin and naringenin, on metabolic syndrome and their mechanisms of action. Advr Nutr.

[B7] Ali BH, Al Za’abi M, Blunden G, Nemmar A (2011). Experimental gentamicin nephrotoxicity and agents that modify it: a mini‐review of recent research. Basic Clin Pharmacol Toxicol.

[B8] Amini N, Sarkaki A, Dianat M, Mard SA, Ahangarpour A, Badavi M (2019a). Protective effects of naringin and trimetazidine on remote effect of acute renal injury on oxidative stress and myocardial injury through Nrf-2 regulation. Pharmacol Rep.

[B9] Amini N, Sarkaki A, Dianat M, Mard SA, Ahangarpour A, Badavi M (2019b). The renoprotective effects of naringin and trimetazidine on renal ischemia/reperfusion injury in rats through inhibition of apoptosis and downregulation of micoRNA-10a. Biomed Pharmaco.

[B10] Amudha K, Pari L (2011). Beneficial role of naringin, a flavanoid on nickel induced nephrotoxicity in rats. Chem Bio Interact.

[B11] Anjaneyulu M, Tirkey N, Chopra K (2003). Attenuation of cyclosporine-induced renal dysfunction by catechin: possible antioxidant mechanism. Ren Fail.

[B12] Araujo M, Welch WJ (2006). Oxidative stress and nitric oxide in kidney function. Curr Opin Nephrol Hypertens.

[B13] Bae EH, Kim IJ, Joo SY, Kim EY, Choi JS, Kim CS, Ma SK, Lee J, Kim SW (2014). Renoprotective effects of the direct renin inhibitor aliskiren on gentamicin-induced nephrotoxicity in rats. J Renin Angiotensin Aldosterone Syst.

[B14] Balakumar P, Rohilla A, Thangathirupathi A (2010). Gentamicin-induced nephrotoxicity: do we have a promising therapeutic approach to blunt it?. Pharmacol Res.

[B15] Bello-Reuss E, Reuss L (1983). Homeostatic and Excretory Functions of the Kidney. The Kidney and Body Fluids in Health and Disease.

[B16] Benzer F, Kandemir F, Ceribasi S, Ozkaraca M, Yildirim N, Ozan S (2012). Chemotherapeutic agent-induced nephrotoxicity in rabbits: protective role of grape seed extract. Int J Pharmacol.

[B17] Bo CJ, Chen B, Jia RP, Zhu JG, Cao P, Liu H, Wu R, Ge YZ, Wu JP (2013). Effects of ischemic preconditioning in the late phase on homing of endothelial progenitor cells in renal ischemia/reperfusion injury. Transplant Proc.

[B18] Bubici C, Papa S, Dean K, Franzoso G (2006). Mutual cross-talk between reactive oxygen species and nuclear factor-kappa B: molecular basis and biological significance. Oncogene.

[B19] Busauschina A, Schnuelle P, Van der Woude F (2004). Cyclosporine nephrotoxicity. Transplant Proc.

[B20] Camargo CA, Gomes-Marcondes MCC, Wutzki NC, Aoyama H (2012). Naringin inhibits tumor growth and reduces interleukin-6 and tumor necrosis factor α levels in rats with Walker 256 carcinosarcoma. Anticancer Res.

[B21] Chandramohan Y, Parameswari CS (2013). Therapeutic efficacy of naringin on cyclosporine (A) induced nephrotoxicity in rats: involvement of hemeoxygenase-1. Pharmacol Rep.

[B22] Chen L, Teng H, Jia Z, Battino M, Miron A, Yu Z, Cao H, Xiao J (2018a). Intracellular signaling pathways of inflammation modulated by dietary flavonoids: The most recent evidence. Crit Rev Food Sci Nutr.

[B23] Chen L, Teng H, Xie Z, Cao H, Cheang WS, Skalicka-Woniak K, Georgiev MI, Xiao J (2018b). Modifications of dietary flavonoids towards improved bioactivity: An update on structure–activity relationship. Crit Rev Food Sci Nutr.

[B24] Choudhury R, Chowrimootoo G, Srai K, Debnam E, Rice-Evans CA (1999). Interactions of the flavonoid naringenin in the gastrointestinal tract and the influence of glycosylation. Biochem Biophys Res Commun Biochem.

[B25] Chtourou Y, Aouey B, Aroui S, Kebieche M, Fetoui H (2016). Anti-apoptotic and anti-inflammatory effects of naringin on cisplatin-induced renal injury in the rat. Chem Biolo Interac.

[B26] Chtourou Y, Gargouri B, Kebieche M, Fetoui H (2015). Naringin abrogates cisplatin-induced cognitive deficits and cholinergic dysfunction through the down-regulation of AChE expression and iNOS signaling pathways in hippocampus of aged rats. J Mol Neurosci.

[B27] Chung J, Nguyen A-K, Henstridge DC, Holmes AG, Chan MS, Mesa JL, Lancaster GI, Southgate RJ, Bruce CR, Duffy SJ (2008). HSP72 protects against obesity-induced insulin resistance. Proc Natl Acad Sci.

[B28] De Brouwere K, Buekers J, Cornelis C, Schlekat CE, Oller AR (2012). Assessment of indirect human exposure to environmental sources of nickel: oral exposure and risk characterization for systemic effects. Sci Total Environ.

[B29] Di Lullo L, Reeves PB, Bellasi A, Ronco C (2019). Cardiorenal syndrome in acute kidney injury. Semin Nephrol.

[B30] Edelman DA, Roggli VL (1989). The accumulation of nickel in human lungs. Environl Health Perspect.

[B31] Ferguson MA, Vaidya VS, Bonventre J (2008). Biomarkers of nephrotoxic acute kidney injury. Toxicol.

[B32] Gelen V, Sengul E, Yildirim S, Atila G (2018). The protective effects of naringin against 5-fluorouracil-induced hepatotoxicity and nephrotoxicity in rats. Iran J Basic Med Sci.

[B33] Gitlitz PH, Sunderman Jr FW, Goldblatt PJ (1975). Aminoaciduria and proteinuria in rats after a single intraperitoneal injection of Ni (II). Toxicol Appl Pharmacol.

[B34] Gülcin I (2012). Antioxidant activity of food constituents: an overview. Archiv Toxicol.

[B35] Gum SI, Cho MK (2013). Recent updates on acetaminophen hepatotoxicity: the role of nrf2 in hepatoprotection. Toxicol Res.

[B36] Huang Y, Li W, Su Z-y, Kong A-NT (2015). The complexity of the Nrf2 pathway: beyond the antioxidant response. J Nutr Biochem.

[B37] Ichimura T, Asseldonk EJ, Humphreys BD, Gunaratnam L, Duffield JS, Bonventre JV (2008). Kidney injury molecule–1 is a phosphatidylserine receptor that confers a phagocytic phenotype on epithelial cells. J Clin Investig J Clin Invest.

[B38] Isik B, Bayrak R, Akcay A, Sogut S (2006). Erdosteine against acetaminophen induced renal toxicity. Mol Cell Biochem.

[B39] Jänicke RU, Sprengart ML, Wati MR, Porter AG (1998). Caspase-3 is required for DNA fragmentation and morphological changes associated with apoptosis. J Biol Chem.

[B40] Jollow D, Mitchell J, Potter W, Davis D, Gillette J, Brodie B (1973). Acetaminophen-induced hepatic necrosis Role of covalent binding in vivo. J Pharmacol Experiment Ther.

[B41] Juan S-H, Chen C-H, Hsu Y-H, Hou C-C, Chen T-H, Lin H, Chu Y-L, Sue Y-M (2007). Tetramethylpyrazine protects rat renal tubular cell apoptosis induced by gentamicin. Nephrol Dial Transplant.

[B42] Kalemci S, Topal Y, Celik SY, Yilmaz N, Beydilli H, Kosar MI, Dirican N, Altuntas I (2015). Silibinin attenuates methotrexate-induced pulmonary injury by targeting oxidative stress. Exper Ther Med.

[B43] Kalogeris T, Baines CP, Krenz M, Korthuis RJ (2012). Cell biology of ischemia/reperfusion injury. Int Rev Cell Mol Biol.

[B44] Kandemir FM, Kucukler S, Caglayan C, Gur C, Batil AA, Gülçin İ (2017). Therapeutic effects of silymarin and naringin on methotrexate‐induced nephrotoxicity in rats: Biochemical evaluation of anti‐inflammatory, antiapoptotic, and antiautophagic properties. J Food Biochem.

[B45] Kimura A, Ishida Y, Hayashi T, Wada T, Yokoyama H, Sugaya T, Mukaida N, Kondo T (2006). Interferon-γ plays protective roles in sodium arsenite-induced renal injury by up-regulating intrarenal multidrug resistance-associated protein 1 expression. Am J Pathol.

[B46] Lee I-C, Kim S-H, Lee S-M, Baek H-S, Moon C, Kim S-H, Park S-C, Kim H-C, Kim J-C (2012). Melatonin attenuates gentamicin-induced nephrotoxicity and oxidative stress in rats. Archiv Toxicol.

[B47] Li P, Wang S, Guan X, Cen X, Hu C, Peng W, Wang Y, Su W (2014). Six months chronic toxicological evaluation of naringin in Sprague–Dawley rats. Food Chem Toxicol.

[B48] Li P, Wang S, Guan X, Liu B, Wang Y, Xu K, Peng W, Su W, Zhang K (2013). Acute and 13 weeks subchronic toxicological evaluation of naringin in Sprague-Dawley rats. Food Chem Toxicol.

[B49] Liu J, Liu Y, Goyer RA, Achanzar W, Waalkes MP (2000). Metallothionein-I/II null mice are more sensitive than wild-type mice to the hepatotoxic and nephrotoxic effects of chronic oral or injected inorganic arsenicals. Toxicol Sci.

[B50] Longley DB, Harkin DP, Johnston PG (2003). 5-fluorouracil: mechanisms of action and clinical strategies. Nat Rev Cancer.

[B51] Martin KR, Appel CL (2009). Polyphenols as dietary supplements: A double-edged sword. Nutr Diet Suppl.

[B52] McDonough RP, Doucette WR (2003). Drug therapy management: an empirical report of drug therapy problems, pharmacists' interventions, and results of pharmacists' actions. J Am Pharm Asso.

[B53] Michalik L, Wahli W (2006). Involvement of PPAR nuclear receptors in tissue injury and wound repair. J Cli Invest.

[B54] Molaei E, Molaei A, Abedi F, Hayes AW, Karimi G (2021). Nephroprotective activity of natural products against chemical toxicants: The role of Nrf2/ARE signaling pathway. Food Sci Nutr.

[B55] Morales AI, Detaille D, Prieto M, Puente A, Briones E, Arévalo M, Leverve X, López-Novoa JM, El-Mir M-Y (2010). Metformin prevents experimental gentamicin-induced nephropathy by a mitochondria-dependent pathway. Kidney Int.

[B56] Nath KA (2006). Heme oxygenase-1: a provenance for cytoprotective pathways in the kidney and other tissues. Kidney Int.

[B57] Negrette-Guzmán M, Huerta-Yepez S, Medina-Campos ON, Zatarain-Barrón ZL, Hernández-Pando R, Torres I, Tapia E, Pedraza-Chaverri J (2013). Sulforaphane attenuates gentamicin-induced nephrotoxicity: role of mitochondrial protection. Evid Based Complement Alternat Med.

[B58] Nejabat M, Kahe H, Shirani K, Ghorbannejad P, Hadizadeh F, Karimi G (2017). Health risk assessment of heavy metals via dietary intake of wheat in Golestan Province, Iran. Hum Ecol Risk Assess.

[B59] Pabla N, Dong Z (2008). Cisplatin nephrotoxicity: mechanisms and renoprotective strategies. Kidney Int.

[B60] Ratliff BB, Abdulmahdi W, Pawar R, Wolin MS (2016). Oxidant mechanisms in renal injury and disease. Antioxid Redox Signal.

[B61] Rehman MU, Tahir M, Ali F, Qamar W, Lateef A, Khan R, Quaiyoom A, Sultana S (2012). Cyclophosphamide-induced nephrotoxicity, genotoxicity, and damage in kidney genomic DNA of Swiss albino mice: the protective effect of Ellagic acid. Mol Cell Biochem.

[B62] Rodríguez V, Plavnik L, de Talamoni NT (2018). Naringin attenuates liver damage in streptozotocin-induced diabetic rats. Biomed Pharmacother.

[B63] Sahu BD, Tatireddy S, Koneru M, Borkar RM, Kumar JM, Kuncha M, Srinivas R, Shyam Sunder R, Sistla R (2014). Naringin ameliorates gentamicin-induced nephrotoxicity and associated mitochondrial dysfunction, apoptosis and inflammation in rats: possible mechanism of nephroprotection. Toxicol appl pharmacol.

[B64] Sharma AK, Bharti S, Ojha S, Bhatia J, Kumar N, Ray R, Kumari S, Arya DS (2011). Up-regulation of PPARγ, heat shock protein-27 and-72 by naringin attenuates insulin resistance, β-cell dysfunction, hepatic steatosis and kidney damage in a rat model of type 2 diabetes. Br J Nutr.

[B65] Shaw JE, Sicree RA, Zimmet PZ (2010). Global estimates of the prevalence of diabetes for 2010 and 2030. Diabetes Res Clin Pract.

[B66] Shoskes DA, Halloran PF (1996). Delayed graft function in renal transplantation: etiology, management and long-term significance. J Urol.

[B67] Sinanoglu O, Yener AN, Ekici S, Midi A, Aksungar FB (2012). The protective effects of spirulina in cyclophosphamide induced nephrotoxicity and urotoxicity in rats. Urology.

[B68] Singh D, Chander V, Chopra K (2004a). Protective effect of naringin, a bioflavonoid on ferric nitrilotriacetate-induced oxidative renal damage in rat kidney. Toxicol.

[B69] Singh D, Chander V, Chopra K (2004b). Protective effect of naringin, a bioflavonoid on glycerol-induced acute renal failure in rat kidney. Toxicol.

[B70] Singh KP, DuMond JW (2007). Genetic and epigenetic changes induced by chronic low dose exposure to arsenic of mouse testicular Leydig cells. Int J Oncol.

[B71] Sugumar E, Kanakasabapathy I, Abraham P (2007). Normal plasma creatinine level despite histological evidence of damage and increased oxidative stress in the kidneys of cyclophosphamide treated rats. Clin Chim Acta.

[B72] Sun X, Zhang B, Hong X, Zhang X, Kong X (2013). Histone deacetylase inhibitor, sodium butyrate, attenuates gentamicin-induced nephrotoxicity by increasing prohibitin protein expression in rats. Eur J.

[B73] Taha A-EA, Khalifa M, El-Salam A, Mohamed I (2018). Naringin prevent cisplatin-induced nephrotoxicity by abrogation of oxidative stress and inflammation in rats. Bull Pharm Sci.

[B74] Tripoli E, La Guardia M, Giammanco S, Di Majo D, Giammanco M (2007). Citrus flavonoids: Molecular structure, biological activity and nutritional properties: A review. Food Chem.

[B75] Tsai Y-J, Tsai T-H (2012). Mesenteric lymphatic absorption and the pharmacokinetics of naringin and naringenin in the rat. J Agric Food Chem.

[B76] Visnagri A, Kandhare AD, Bodhankar SL (2015). Renoprotective effect of berberine via intonation on apoptosis and mitochondrial-dependent pathway in renal ischemia reperfusion-induced mutilation. Renal Fail.

[B77] Wang GG, Lu XH, Li W, Zhao X, Zhang C (2011). Protective effects of luteolin on diabetic nephropathy in STZ-induced diabetic rats. Evid Based Complement Alternat Med.

[B78] Wang N, Zhou Y, Jiang L, Li D, Yang J, Zhang C-Y, Zen K (2012). Urinary microRNA-10a and microRNA-30d serve as novel, sensitive and specific biomarkers for kidney injury. PloS One.

[B79] Wolf G, Ziyadeh FN (2007). Cellular and molecular mechanisms of proteinuria in diabetic nephropathy. Nephron Physiol.

[B80] Yadav M, Sehrawat N, Singh M, Upadhyay SK, Aggarwal D, Sharma AK (2020). Cardioprotective and hepatoprotective potential of citrus flavonoid naringin: Current status and future perspectives for health benefits. Asian J Biol Sci.

[B81] Zhang J, Gao W, Liu Z, Zhang Z, Liu C (2014). Systematic analysis of main constituents in rat biological samples after oral administration of the methanol extract of fructus Aurantii by HPLC-ESI-MS/MS. Iranian J Pharm Res.

[B82] Zhang J, Yang S, Li H, Chen F, Shi J (2017). Naringin ameliorates diabetic nephropathy by inhibiting NADPH oxidase 4. Eur J Pharmacol.

